# Retraction: Dickkopf 2 serves as a novel therapeutic target and prognostic biomarker in acute myeloid leukemia targeted by evodiamine

**DOI:** 10.3389/fmed.2026.2023213

**Published:** 2026-10-28

**Authors:** Frontiers Editorial Office

The publisher retracts the 30 April 2026 article cited above.

Following publication, concerns were raised regarding the integrity of the data in the manuscript. The authors failed to provide a satisfactory explanation during the investigation, which was conducted in accordance with Frontiers’ policies.

This retraction was approved by the Chief Editors of Frontiers in Medicine and the Chief Executive Editor of Frontiers. The authors received a communication regarding the retraction and had a chance to respond. This communication has been recorded by the publisher.

Frontiers would like to thank Sholto David for contacting the journal regarding the published article.

